# The Mitogen-Activated Protein Kinase *CgMK1* Governs Appressorium Formation, Melanin Synthesis, and Plant Infection of *Colletotrichum gloeosporioides*

**DOI:** 10.3389/fmicb.2017.02216

**Published:** 2017-11-10

**Authors:** Puhuizhong He, Yonglin Wang, Xiaolian Wang, Xiaolin Zhang, Chengming Tian

**Affiliations:** College of Forestry, Beijing Forestry University, Beijing, China

**Keywords:** *Colletotrichum gloeosporiodes*, MAPK, appressorium formation, virulence, penetration

## Abstract

The fungus *Colletotrichum gloeosporiodes* infects plant hosts with a specialized cell called an appressorium, which is melanized and required for plant cell wall penetration. Here, we show that the mitogen-activated protein kinase *CgMK1* governs appressorium formation and virulence in the poplar anthracnose fungus *C. gloeosporioides*. Deletion of *CgMK1* impairs aerial hyphal growth and biomass accumulation, and *CgMK1* is responsible for the expression of melanin biosynthesis-associated genes. *CgMK1* deletion mutants are unable to form appressorium and lose the capacity to colonize either wounded or unwounded poplar leaves, leading to loss of virulence. We demonstrate that the exogenous application of cAMP fails to restore defective appressorium formation in the *CgMK1* deletion mutants, suggesting that *CgMK1* may function downstream or independent of a cAMP-dependent signal for appressorium formation. Moreover, *CgMK1* mutants were sensitive to high osmosis, indicating that *CgMK1* plays an important role in stress response. We conclude that *CgMK1* plays a vital role in regulating appressorium formation, melanin biosynthesis, and virulence in *C. gloeosporiodes*.

## Introduction

Various genes in phytopathogenic fungi are involved in certain signaling pathways associated with the recognition of chemical substances from the host and in response to other extracellular stimuli. The mitogen-activated protein kinase (MAPK) signaling pathway is a downstream cellular signaling event that is remarkably conserved and mainly consists of MAPK cascades, including three-tiered protein kinase modules by which phosphorylation sites of tyrosine and threonine deliver signals, ultimately activating the downstream components of the pathway (Marshall, 1994; Seger and Krebs, 1995; Hamel et al., 2012). MAPK signaling pathways are divided into five categories that are classified according to the different functions of *Saccharomyces cerevisiae*, including conditioning the response of mating pheromones, controlling homeostasis under high osmolarity stress, and maintaining cell wall integrity (Herskowitz, 1995). The interplay of these signaling pathways in certain external stress conditions has previously been illustrated (Baltanás et al., 2013). Among the five types of MAPK signaling pathways, the functions of a Fus3/Kss1-related gene in phytopathogenic fungi include, in addition to filamentous growth, a vital role in virulence and in host surface colonization and the formation of infection structures, as well as penetration and development into the cell tissues of the host.

Fus3/Kss1 orthologs are denominated as “pathogenic MAPKs,” which are vital genes that are significant for infection structure differentiation and various processes in pathogenic fungi. Increasing evidence has indicated that the sensor genes orchestrate virulence via Fus3/Kss1 orthologs, such as *SHO1* and *MSB2* (Leroch et al., 2015). Various components that converge downstream of fus3/Kss1 have also been identified, such as STE12 (Park et al., 2002) and SLF1 (Li et al., 2011). Some genes associated with other signaling pathways are also implicated in fus3/kss1-dependent patterns such as the inactivation of the TOR pathway (Marroquin-Guzman and Wilson, 2015), as well as the thioredoxin-associated gene, *TRX2* (Wang et al., 2016). Previous studies have shown that the activation of fus3/kss1-associated genes is necessary for the development of a unique infection structure for endangering hosts in phytopathogenic fungi. For instance, in *Magnaporthe oryzae*, deletion of PMK1 (Fus3/Kss1 ortholog) disrupts the capacity of appressoria differentiation, thereby resulting in non-pathogenicity (Xu and Hamer, 1996). Additionally, strains lacking PMK1 are also prevented from attacking the host via wounded tissues, suggesting that PMK1 is vital for various invasive stages of fungal attack toward the host. Subsequently, ortholog analysis has demonstrated that Fus3/Kss1 is essential for pathogenicity in phytopathogens, not only in appressorium-forming fungi, such as *CMK1* in *Colletotrichum orbiculare* (Takano et al., 2000) and *ChMK1* in *C. higginsianum* (Wei et al., 2016), but also in non-classical appressorium-forming fungi such as *VMK1* in *Verticillium dahliae* (Rauyaree et al., 2005) and *CsFUS3* in *Bipolaris sorokiniana* (Leng and Zhong, 2015). Indeed, these MAPKs have been verified to be closely associated with the infection morphology and pathogenicity of fungi.

Anthracnose, a typical poplar foliage disease generated by the hemibiotrophic ascomycete fungus *Colletotrichum gloeosporioides*, is highly prevalent in northeast China and results in substantial economic losses. During pathogenesis process, an infectious structure characterized by the appearance of dome-like structures called forming at the hyphal apex (Kleemann et al., 2008). Previous studies have explored the significance of MAPK components and the cAMP/PKA pathway in appressorium formation in *C. gloeosporioides.* The functions of certain MAPK modules have been reported, such as the deletion of *Cgl-SLT2* results in deficient sporulation, disruption of appressorium formation, and a reduction in pathogenicity in mango (Yong et al., 2013). *CgMEK1* deletion mutants exhibit defective in appressoria development and the loss of virulence (Kim et al., 2000). Moreover, *CgPKAC*, a cAMP pathway-related protein kinase in *C. gloeosporioides*, is an imperative gene in virulence and appressorium formation (Priyatno et al., 2012). Together, these findings could facilitate the elucidation of the molecular mechanism underlying appressorium formation. However, details investigations on the signaling cascades in *C. gloeosporioides* are not well-studied to date.

We therefore aimed to explore the multiple functions of *CgMK1*. Here, we demonstrated that *CgMK1* (an ortholog of Fus3/Kss1) in *C. gloeosporioides* plays an important role in appressorium formation and pathogenesis. The results also indicated that *CgMK1* affects aerial hyphal growth and governs the expression of melanin production-related genes. Furthermore, based on the exogenous application of cAMP, we reveal that *CgMK1* may function downstream or independent of a cAMP-dependent signal in appressorium formation.

## Materials and Methods

### Strains and Growth Tests

The wild-type of *C. gloeosporioides* is CFCC80308, isolated from *Populus × beijingensis*. The wild-type and its derivatives were cultured in complete medium (CM) or on potato dextrose agar (PDA) plates at 25°C with day/night cycles as previously described (Xu et al., 2016). To assay for defects in stress responses, the size and morphology of the colonies were measured with cultures grown on CM with 1.2 M NaCl, 1 M sorbitol, Congo Red (100 μg/ml), or Calcofluor (60 μg/ml). Statistical analyses of all data were performed by using the Duncan’s range test of SPSS20.0. The *P*-value < 0.05 was considered as statistically significant. All assays were performed in triplicate, in three independent experiments.

### Bioinformatics Analysis of *CgMK1*

The sequence of *CgMK1* was retrieved from the *C. gloeosporioides* genome database^1^. A BLASTp search on the National Center for Biotechnology Information (NCBI) website was used to find all annotated Fus3 genes from other fungal genomes. The InterPro^2^ was used to predict domain of *CgMK1*. Amino-acid sequences of *CgMK1* and Fus3/Kss1 homologs from other fungi were aligned by the ClustalX 2.1. For homology analysis, we constructed a phylogenetic tree in MEGA 7.0 with 1000 bootstrap replicates and the neighbor-joining method as previously described (Sun et al., 2016).

### Targeted Gene Knockout and Complementation

The *CgMK1* gene replacement constructs were established using the split-marker gene replacement strategy. To construct the upstream (∼1.2 kb) and downstream (∼1.4 kb) flanking sequences of *CgMK1*, the primers CgMK1-5Ffor, CgMK1-5Frev, CgMK1-3Ffor, and CgMK1-3Frev were used for the 5′-flanking sequence and the 3′-flanking sequence, respectively. The next step required fusing the fragments and two-thirds of the hygromycin cassette by overlapping PCR using the primers CgMK1-5Ffor/HY-R and YG-F/CgMK1-3Frev, respectively. The *CgMK1* gene replacement and transformation methods were performed as described in a previous study (Xu et al., 2016). The PCR assays were used to confirm whether the *CgMK1* gene had been replaced using the primers External-CgMK1for/External-CgMK1rev and RT-CgMK1for/RT-CgMK1rev. To analyze homologous recombination events in the transformants, southern blotting was performed to confirm the deletion of *CgMK1* with the DIG High PrimeDNALabeling and Detection Starter Kit I in conformity to the manufacturers’ protocol (Roche, Germany). *Sca*I was used to digest the genomic DNA of wild-type and transformants. The probe amplified with primers ProbeHPHfor, ProbeHPHrev from HPH and the primers ProbeCgMK1for, ProbeCgMK1rev from the *C. gloeosporioides* CFCC80308 and labeled with DIG primer (**Table 1**).

**Table 1:** PCR primers used in this study.

Primer name	Sequence	Use in this study
CgMK1-5Ffor	CTTACCTGGCCTCGTCGTTT	5F flanking sequence
CgMK1-5Frev	GATACTACTTCGGGCGGAGC	
CgMK1-3Ffor	TCCCATTCCGGAGGAGTTCT	3F flanking sequence
CgMK1-3Frev	CGCTTCAAAGCCTTCCCCTA	
External-CgMK1for	CACCGACCAAGGCAATTGTG	External sequence used for validation of mutant
External-CgMK1rev	GCATTGGTGTGCCGTTCTTT	
CgMK1-Compfor	CGTCCTTTTGCGCACCTTAC	Complementary sequence
CgMK1-Comprev	TAACCTCGAAGACCCTTGCG	
Promoter + CgMK1rev	CCGCATAATCTCCTGGTAAA	Complementary sequence without stop codon
Promoter + CgMK1 + eGFPrev	ACGCTCTTTTCTCTTAGGTTTA	Complementary and sublocation sequence cassette
RT-CgMK1for	TGCTTAGTGGAAAGCCCCTG	qRT-PCR of CgMK1
RT-CgMK1rev	AGCGAATGTACTCTCTGGCG	
Cg18S-up	GTGAGGCCCTCAAAGGTAGTGG	qRT-PCR of Cg18S
Cg18S-down	GGATCCCAGTGCGAGACGT	
ProbeHPHfor	AAAGAACGGCACACCAATGC	Probe HPH sequence used for hybridization
ProbeHPHrev	ATGAGAGAAACGACCAGGCG	
ProbeCgMK1for	GCGAAGAATCTCGTGCTTTC	Probe CgMK1 sequence used for hybridization
ProbeCgMK1rev	GATGTTGGCGACCTCGTATT	
YG-F	GATGTAGGAGGGCGTGGATATGTCCT	The 2/3rd portion of the hygromycin cassette
HY-R	GTATTGACCGATTCCTTGCGGTCCGAA	
Hygromycinfor	CGCCAGGGTTTTCCCAGTCACGAC	hygromycin cassette
Hygromycinrev	AGCGGATAACAATTTCACACAGGA	

The fragment for complementing the *CgMK1* deletion strains, which contains the entire *CgMK1* coding region, its native promoter, and terminator regions, was PCR-amplified using primers CgMK1-Compfor/CgMK1-Comprev, and inserted into a modified pBC-phleo vector conferring phleomycin resistance (provided by FGSC). After harvesting the transformants, we selected the strain from the TB3 medium (with 100 μg/mL phleomycin) and confirmed the results using RT-PCR.

### Generation of the *CgMK1/CgMK1::eGFP* Fusion Construct

To explore the subcellular location of *CgMK1*, the sequence was amplified from the wild-type strain using the primers CgMK1-5Ffor/promoter + CgMK1rev, CgMK1-5Ffor/promoter + CgMK1 + eGFPrev and a pKD6-GFP plasmid. The resulting DNA product was then transferred into the protoplasts of the Δ*CgMK1-13* mutant. Upon harvesting the transformants, we selected the strain from TB3 medium (with 100 μg/mL phleomycin), examined the eGFP signals, and confirmed using qPCR. These tests were conducted as previously described (Xu et al., 2016).

### Quantitative RT-PCR Assays

To obtain whole RNA of the vegetative mycelia, TRIzol reagent (Invitrogen) was used to isolate RNA from the conidia of the wild-type and transformants. The RNA was purified with the PureLink RNA Mini Kit (Ambion). The RNA products were incubated with DNase I (TaKaRa) to reduce DNA contamination. For the quantitative real-time PCR (qRT-PCR) assays, Oligo-DT and SuperScript III reverse transcriptase (Invitrogen) were used to transcribe the mRNA into cDNA. The qRT-PCR reactions were conducted to analyze the transcript levels. The *Cg18S* gene served as an internal reference in this study. The 2^-ΔΔCT^ method was used to analyze gene expression levels of the genes (Livak and Schmittgen, 2001). These tests were carried out as previously described (Xu et al., 2016). Quantitative real-time PCR was performed in triplicate with three independent biological experiments. All primers used in this study are listed in **Table 1**.

### Conidiation and Appressorium Formation Tests

For the conidiation assays, we selected 5 mm diameter mycelial plugs from the edge of a 3-days-old colony that had been placed onto PDA medium and grown at 25°C for 7 days. Conidial suspensions from each strain were selected from the PDA plates after applying 5 mL of sterile water. Conidial suspensions were placed at 25°C for 12 h on a GelBond membrane to allow germination and growth. The suspensions were monitored, and the colony-forming efficiency was assessed under the microscope.

Equal numbers of spores were placed on the hydrophobic surface of the GelBond film (Lonza) at 28°C for 12 h to examine appressoria formation. The appressoria from germinated spores were counted. At least 200 conidia were surveyed in experiments. The rates of conidiation and appressorium formation assays were performed in triplicate, in three independent experiments.

### Pathogenicity Assays

To test the virulence, approximately 3-week-old detached leaves of *Populus × beijingensis* were incubated with 20 μL conidium suspension (10^5^ conidia/mL) at 25°C. Sufficient moisture was maintained, and the morphology and size of the lesions were observed and measured at 4–5 dpi. For the wound-inoculation assays, the conidia of the strain were placed on the wounded sites of the poplar leaves, and the morphology and size of the lesions were observed and measured at 2–3 dpi. At least three virulence experiments were performed, with seven replicates each.

## Results

### Deletion of *CgMK1* Impairs Aerial Hyphae Growth

We explored a gene in the *C. gloeosporioides* genome database (JGI) by BLASTp search that displayed high identity with PMK1 of *M. oryzae*, thus designated as *CgMK1*. *CgMK1* contained the protein kinase domain with a threonine and tyrosine phosphorylation site, and displayed high identity with Fus3/Kss1 from *C. orbiculare* (99.4%), *V. dahliae* (98.0%), *Botrytis cinerea* (94.6%), and *Fusarium graminearum* (98.6%) (**Supplementary Figure S1A**). Furthermore, phylogenetic analysis demonstrated that *CgMK1* is homologous to Fus3/Kss1 MAPKs of other fungi and the Fus3/Kss1 is highly conserved among fungi (**Supplementary Figure S1B**).

To investigate the role of *CgMK1* in *C. gloeosporioides*, *CgMK1* deletion mutants (Δ*CgMK1-8*, Δ*CgMK1-13*) were obtained by replacing the wild-type *CgMK1* gene with a hygromycin cassette (**Supplementary Figure S2**). For mutant complementation, we reintroduced the wild-type *CgMK1* into the Δ*CgMK1-13* mutant to produce the complementation strain (Δ*CgMK1/CgMK1*; **Supplementary Figure S2E**).

**FIGURE 1:**
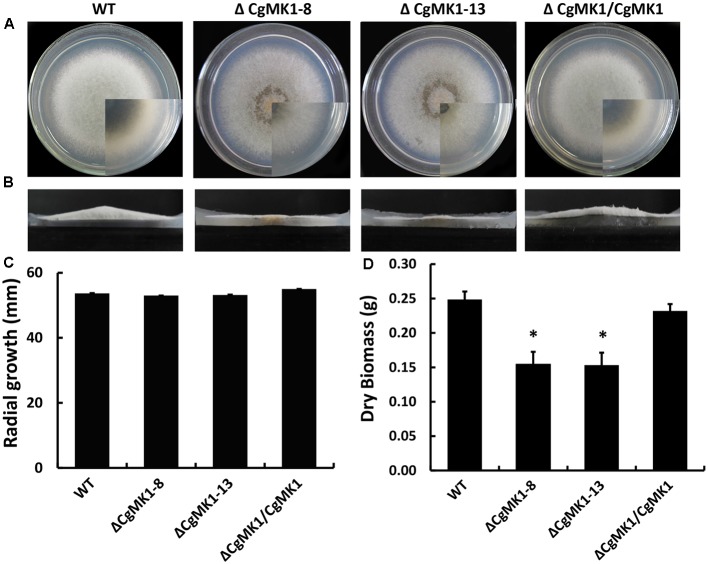
Loss of *CgMK1* impairs aerial hyphae growth. **(A)** The *CgMK1* mutants (Δ*CgMK*1-8 and Δ*CgMK*1-13), the wild-type (WT), and complement (Δ*CgMK1/CgMK1*) were inoculated on PDA plates at 25°C for 5 days. **(B)** Aerial hyphae growth is reduced in the *CgMK1* deletion mutants. Colony side elevations of the indicated strains placed on PDA solid culture medium. **(C)** Relative mycelial growth rate of the strains placed on PDA plates after 4 days. **(D)** Relative dry biomass of the strains in liquid CM after 5 days. Error bars represent the standard deviations based on three independent replicates with three technical replicates each. The data were performed by using the Duncan’s range test. The asterisk indicates significant difference at *P* = 0.05.

While the Δ*CgMK1* mutants showed no obvious defects of growth rate on potato dextrose agar (PDA), aerial hyphal growth was remarkably reduced (**Figures 1A,B**). Likewise, radial growth of the Δ*CgMK1* mutants on solid media showed no difference to the wild-type, but biomass production in the liquid was apparently reduced (**Figures 1C,D**). These results imply that *CgMK1* is essential to aerial hyphal growth.

### Deletion of *CgMK1* Causes the Repression of Melanin Biosynthesis Genes

**FIGURE 2:**
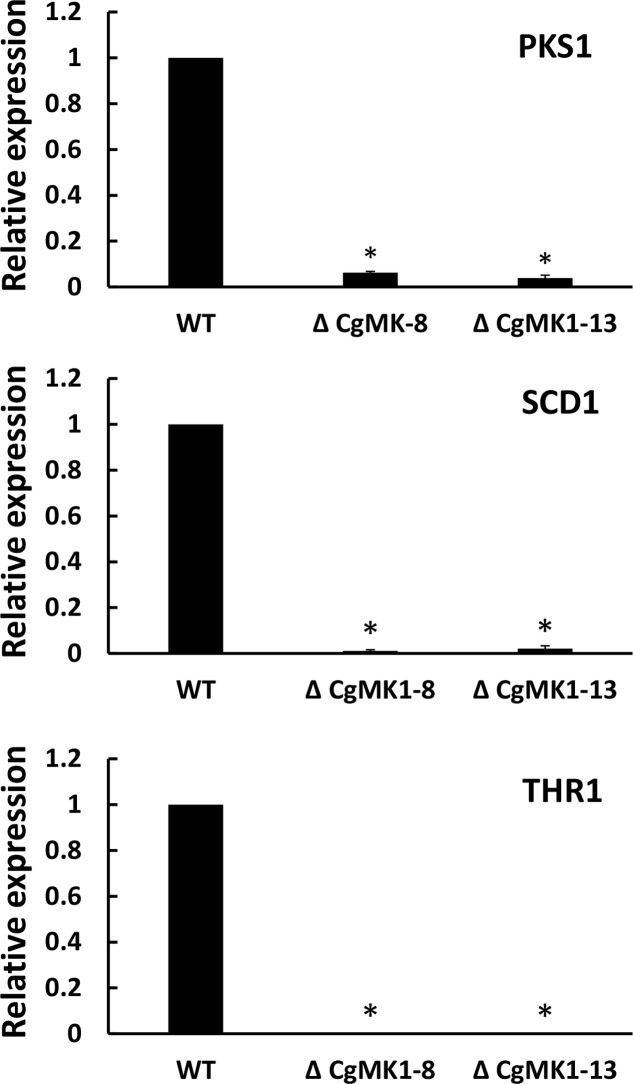
Knockout of *CgMK1* affects the expression of three melanin biosynthesis-related genes. RT-PCR was used for analyzing the transcript levels of the Δ*CgMK1* mutants and wild-type cultured on PDA for 4 days. Transcript levels of *PKS1*, *SCD1*, and *THR1* are expressed in Δ*CgMK1* mutants relative to those of the wild-type. The *Cg18S* gene served as an internal reference for the analysis. Error bars represent the standard deviations based on three independent replicates with four technical replicates each. The data were performed by using the Duncan’s range test. Asterisks mean statistically significant differences at *P* = 0.05.

Because the deletion of *CgMK1* clearly affected melanin deposition in the vegetative hyphae, we then investigated whether the melanin defect of the Δ*CgMK1* mutants could result from the downregulation of melanin biosynthesis genes. To verify the hypothesis, we identified key genes participated in melanin biosynthesis in *C. gloeosporioides* using comparative genomics. Here, three genes associated with melanin production via the dihydroxy naphthalene (DHN) pathway were identified as follows, β-ketoacyl synthase (PKS1), scytalone dehydratase (SCD1), and trihydroxy naphthalene reductase (THR1). The transcript levels of these three genes were measured through quantitative real-time PCR method to compare their expression between wild-type and Δ*CgMK1* mutant. The 2^-ΔΔCT^ method of data statistics showed that the transcription levels of the melanin biosynthesis genes were strongly repressed in the Δ*CgMK1* mutants. The relative expression levels of these genes in the Δ*CgMK1* mutants were at least 50 fold lower gene transcript abundance compared to that in the wild-type strain (**Figure 2**). These results thus indicate that *CgMK1* plays a key role in controlling melanin production.

### *CgMK1* Is Necessary for Appressorium Formation

**FIGURE 3:**
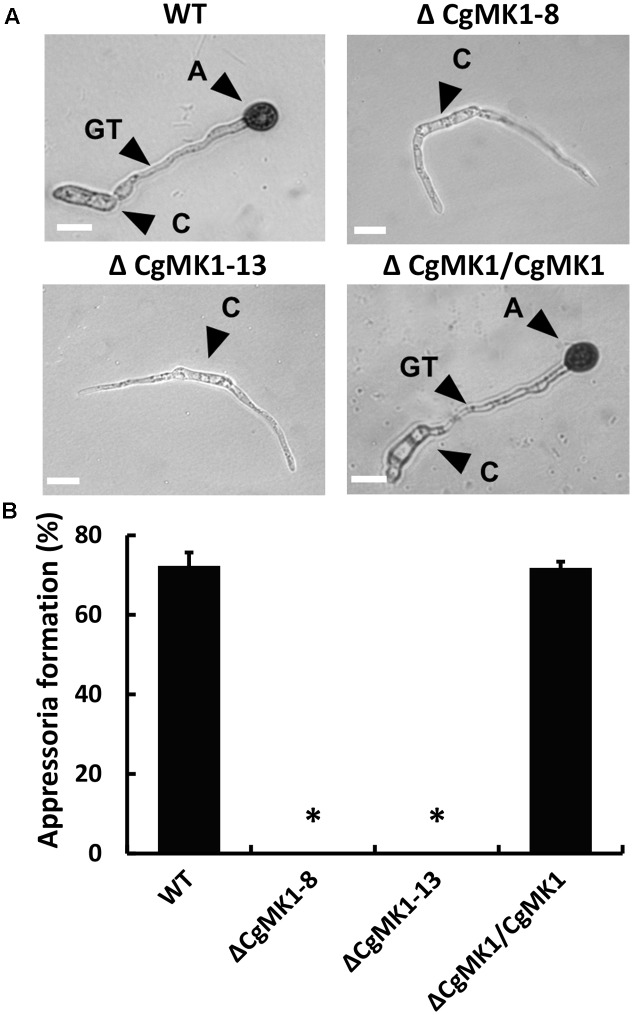
*CgMK1* is essential for appressorium formation. **(A)** The conidial suspension from WT, Δ*CgMK1* mutants, and the Δ*CgMK1/CgMK1* strains were cultured on the hydrophobic surface of the GelBond membrane at 25°C and were observed at 12 h. A: appressorium; C: conidium; GT: germ tube. Scale bar: 10 μm. **(B)** Bar chart showing the appressoria formation rate percentage on the GelBond membrane. The value represents the mean of the three tests. Error bars represent the standard deviations based on three independent replicates with three technical replicates each. The data were performed by using the Duncan’s range test. Asterisks mean statistically significant differences at *P* = 0.05.

We also examined the role of *CgMK1* in appressorium formation and conidia germination using a GelBond membrane. Firstly, we determined that the Δ*CgMK1* mutant and the wild-type exhibit a similar conidia germination phenotypes (data not shown), though the germ tube of the Δ*CgMK1* mutants continued to lengthen instead of forming appressoria at 12 hpi (**Figure 3A**). Based on the data statistics, about 70% of the appressoria were produced in the wild-type strain and Δ*CgMK1/CgMK1*; however, none were produced by the Δ*CgMK1* mutants (**Figure 3B**). These findings demonstrate that *CgMK1* is required for appressoria development.

### Exogenous cAMP Does Not Restore the Appressoria Defective of Δ*CgMK1* Mutants

**FIGURE 4:**
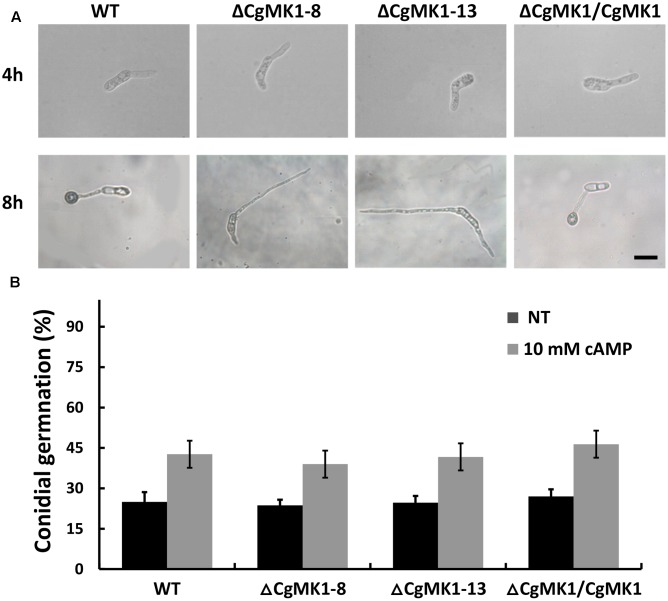
Exogenous application of cAMP fails to restore appressoria formation in the *CgMK1* mutants. **(A)** Conidial germination and appressoria phenotype induced by addition of 10 mM cAMP on the hydrophobic surface of GelBond membrane. Scale bar: 15 μm. **(B)** The germination rate of the strains on the hydrophilic surface of the GelBond membrane in the presence or absence of 10 mM cAMP at 25°C for 4 h. Error bars represent the standard deviations based on three independent replicates with three technical replicates each.

Previous studies have shown that the extracellular application of cAMP induces appressorium formation on hydrophilic surfaces, indicating that the cAMP-dependent signal is important for appressorium development. Here, the application of extracellular cAMP to the Δ*CgMK1* mutants induced conidia germination in a similar manner as in the wild-type, but exogenous cAMP was unable to recover the appressorium formation defect in the Δ*CgMK1* mutants (**Figure 4**). This suggests that *CgMK1* controls the formation of appressoria in a cAMP-independent manner or functions downstream of cAMP signaling.

### Disruption of *CgMK1* Results in a Loss of Virulence

**FIGURE 5:**
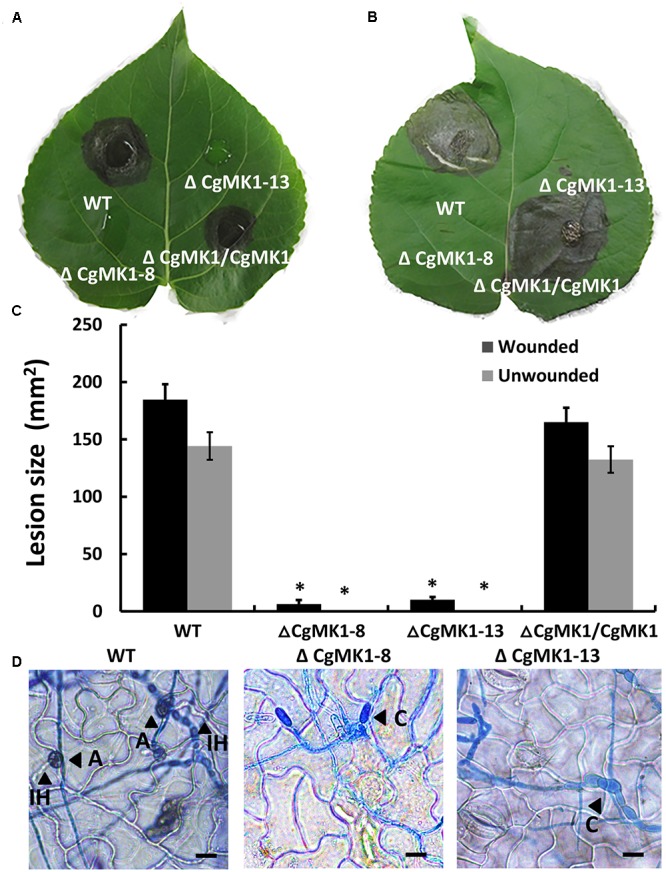
The *CgMK1* deletion mutants lose pathogenicity on poplar leaves, indicating that CgMK1 governs virulence. **(A)** Spores (10^5^/mL) from WT, Δ*CgMK1* mutants and the Δ*CgMK1/CgMK1* were inoculated on the detached leaves of 1-year-old poplar seedlings. Ten healthy intact poplar leaves were used in each independent experiment with three replicates. The images were captured at 7 dpi. **(B)** Spores (10^5^/mL) of WT, Δ*CgMK1* mutants and the Δ*CgMK1/CgMK1* were inoculated on detached leaves of 1-year-old poplar seedlings. Ten poplar leaves were wounded and were used in each independent experiment with three replicates. The images were captured at 7 dpi. **(C)** Quantification of lesion sizes caused by the strains in each independent experiment. Values represent the mean of three repetitions. The data were performed by using the Duncan’s range test. Asterisks mean statistically significant differences at *P* = 0.05. **(D)** Infection hyphae development of the strains on the detached poplar leaves at 48 hpi. Invasive hyphae were treated with lactophenol aniline blue. A: appressorium; C: conidium; IH: invasive hyphae. Scale bar: 10 μm.

The Δ*CgMK1* mutants lost the capability to form appressoria on artificial hydrophobic surfaces. To determine whether this results in defective pathogenicity, the strains were inoculated on detached but intact poplar (*Populus × beijingensis*) leaves. At 7 dpi, the Δ*CgMK1* mutants appeared to completely lose virulence compared to the distinct lesion symptoms of the leaves placed with the Δ*CgMK1/CgMK1* and wild-type (**Figures 5A,C**). Since the Δ*CgMK1* mutants could not form appressoria, the wounded poplar leaves were inoculated with the above strain as well. Similar to the symptoms of the intact leaves, the wild-type strain and Δ*CgMK1/CgMK1* caused typical disease symptoms; in contrast, the Δ*CgMK1* mutants failed to infect the wounded leaves (**Figures 5B,C**). Although the wild-type and Δ*CgMK1/CgMK1* inoculated on the leaf surfaces produced masses of invasive hyphae by 2 dpi, the Δ*CgMK1* mutants did not (**Figure 5D**). Microscopic observations showed that the Δ*CgMK1* mutants extended the germ tube but hardly penetrated into the plant cell. Conversely, in the wild-type, the majority of the conidia developed melanized appressoria, which invaded the epidermal cells and then formed swollen invading hyphae (**Figure 5D**).

**FIGURE 6:**
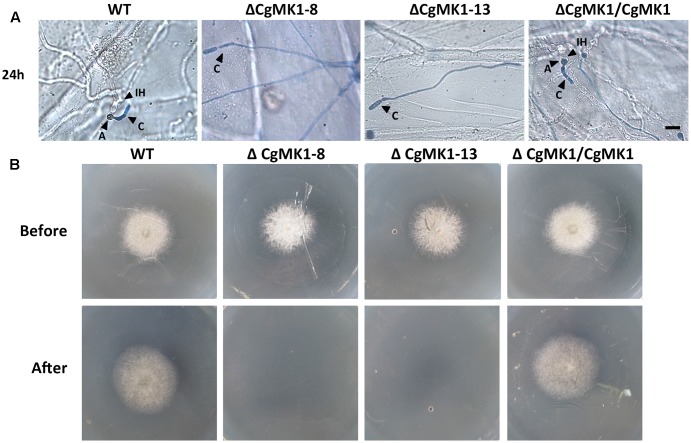
Assays for penetration and colonization defects in the *CgMK1* mutants. **(A)** Onion epidermal cell penetration assay of the Δ*CgMK1* mutants. The assay was performed by inoculating conidial suspensions from the indicated strains. Examination under a light microscope was performed after aniline-blue staining. A: appressorium; C: conidium; IH: invasive hyphae. Scale bar: 10 μm. **(B)** Cellophane membranes penetration assay was performed by the conidial suspensions from strains. Colonies of the indicated strains grown on PDA plates covered with a cellophane membrane (before). Then the medium was removed the cellophane membrane and placed for additional days (after). Photos in the first row were taken at 3 dpi, the second row were taken at 5 dpi that showed growth of strains after penetration from cellophane membrane.

We also performed a penetration assay on onion epidermal cells and cellophane membranes. The results showed that the Δ*CgMK1* mutants produced massive amounts of hyphae on the onion epidermis, but very few hyphae could penetrate into the onion epidermal cells. Nevertheless, the wild-type and Δ*CgMK1/CgMK1* strains infected the epidermal cells and expanded into the epidermal tissues at 24 hpi (**Figure 6A**). We also found that, in contrast to the Δ*CgMK1/CgMK1* and wild-type, the Δ*CgMK1* mutants failed to penetrate the cellophane membranes and were then unable to grow (**Figure 6B**). Collectively, the Δ*CgMK1* mutants lost virulence due to their failure to penetrate into the plant tissues.

### Subcellular Localization of the *CgMK1*

**FIGURE 7:**
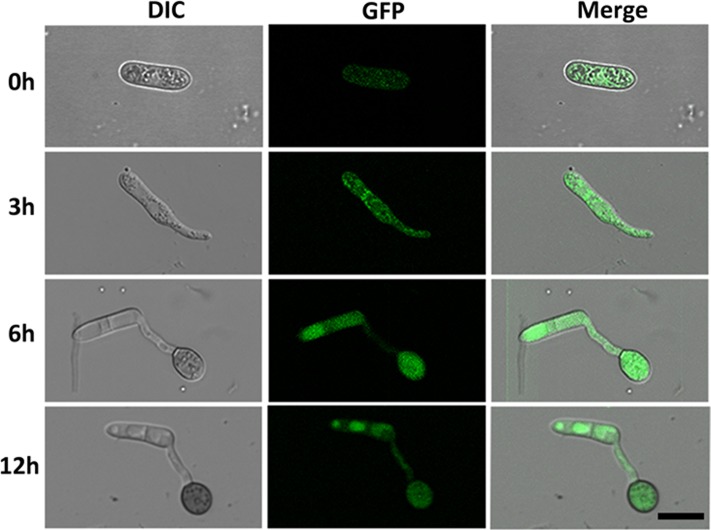
Subcellular localization of *CgMK1* in appressorium formation. Fluorescence of *CgMK1* was detected by C-terminal eGFP fusion under its promoter. Green fluorescence was detected during the stages of germination and appressorium formation on the hydrophobic surface of the GelBond membrane. Scale bar: 10 μm.

To investigate the cellular localization of *CgMK1* in *C. gloeosporioides*, we generated a fragment containing the *CgMK1* and enhanced green fluorescent protein (eGFP), hereby designated as *CgMK1_C::eGFP*. The *CgMK1_C::eGFP* and phleomycin resistance cassette were introduced into Δ*CgMK1-13* protoplasts by co-transformation. GFP signals were detected under the active expression of the native promoter and whole *CgMK1* sequence. In our study, the fluorescence was detected at different stages of conidium, germinating conidia, germ tube, and appressorium formation (**Figure 7**) Moreover, the expression was relatively higher in the mature appressoria than the other stages. These results indicate that *CgMK1* is induced during appressorium formation.

### *CgMK1* Is Involved in the Response to High Osmotic Stress

**FIGURE 8:**
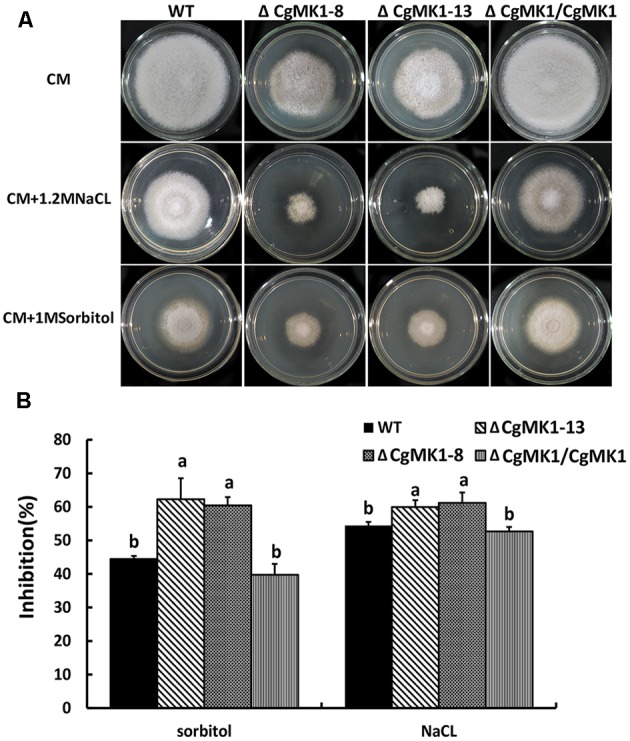
*CgMK1* in response to osmotic stresses. **(A)** Colony morphology of the wild-type strain, Δ*CgMK1* mutants (Δ*CgMK1-8*, Δ*CgMK1-13*) and complemented strain after 3 days of growth on CM or CM containing 1 M Sorbitol, 1.2 M NaCl. **(B)** The bar chart showed the colony diameter of wild-type, mutants and complementation strain under different chemical stresses. Data sets were calculated from the picture **(A)**. “a” and “b” indicate a significant difference between wild-type, mutant and complementation strain at *P* = 0.05 according to Duncan’s range test.

To determine the function of *CgMK1* in stress responses, we observed the wild-type, Δ*CgMK1* and Δ*CgMK1/CgMK1* strains on complete medium (CM) with NaCl, sorbital, calcofluor white (CFW) and Congo red (CR). The Δ*CgMK1* mutants showed sensitivity to 1.2 M NaCl, 1 M Sorbitol (**Figure 8A**). We also tested the growth rate of the strains on plates containing NaCl and sorbitol. The rate of growth inhibition of Δ*CgMK1* mutants when grown on CM containing NaCl and sorbitol was higher than that of wild-type and Δ*CgMK1/CgMK1* strain, respectively (**Figure 8B**). On the other hand, the Δ*CgMK1* mutants showed no obvious difference from wild-type and Δ*CgMK1/CgMK1* strain in growth in response to CFW and CR (**Supplementary Figure S3**). These results suggest that *CgMK1* is involved in the response to high osmotic stress, but not important in the maintenance of cell wall integrity.

## Discussion

Here, we examined the functions of *CgMK1* in appressorium formation and virulence of poplar anthracnose fungus, *C. gloeosporioides*. The results demonstrated that *CgMK1* plays key roles in appressorium formation, melanin production, and plant infection.

Fungal MAPK cascades play key roles in governing fungal development and signaling fungal responses to a variety of stress stimuli. In search of the component of MAPK, Fus3/Kss1-type MAPK was found to be required for fungi to attack plant tissues. Studies on the Fus3/Kss1-type MAPK in pathogenic fungi has revealed the importance in the production of infection structures (Zhao and Xu, 2007; Zhao et al., 2007). In pathogenic fungi, appressoria are special infection structures that impart high turgor pressure and excrete plant cell degradation enzymes that aid the penetration of the infection peg into plant cells (Mendgen et al., 1996; van Kan, 2006). Previous studies have demonstrated that many conserved signal modules are involved in appressorium formation and pathogenicity, at least including small GTPases (Fukada and Kubo, 2015; Xu et al., 2016) and the MAPK pathway (Hamel et al., 2012). In *C. gloeosporioides*, loss of *CgRhoB* resulted in shorter germ tubes and enhanced appressoria formation after germination on the hydrophobic surface. However, the present study demonstrated that *CgMK1* is crucial for appressorium formation rather than conidia germination in *C. gloeosporioides*. The Δ*CgMK1* mutants exhibited no obviously defective in conidia germination, indicating that *CgMK1* does not contribute to conidia germination, which differs from *C. orbiculare* (Takano et al., 2000; Xiong et al., 2015). Subcellular localization of the *CgMK1* showed that *CgMK1* is induced during appressorium formation.

The *C. gloeosporioides* strain lacking the *CgPKAC* gene displayed delayed appressorium formation (Priyatno et al., 2012). The CMK1 cooperated with cAMP to control appressorium formation and germination in *C. orbiculare* (Takano et al., 2000). The PMK1 deletion mutants failed to develop appressoria on Teflon membranes in *M. oryzae*, and formed swollen germ tubes when exogenous cAMP was applied to the hydrophilic surfaces (Xu and Hamer, 1996). The results indicated that cAMP is necessary for appressorium formation and functions upstream of MAPK in appressoria-forming fungi. To explore the reason why the Δ*CgMK1* mutants failed to form the appressorium, we also revealed the function of exogenous cAMP in Δ*CgMK1* mutants. The results showed that exogenous cAMP application does not restore the defect of appressorium formation in the Δ*CgMK1* mutants. This suggested that *CgMK1* controls the formation of appressoria in a cAMP-independent manner or functions downstream of cAMP signaling.

Appressorium melanization is vital for the high turgor pressure necessary for the invasion and growth of appressoria-forming pathogens (Howard and Ferrari, 1989). In *C. orbiculare* (Takano et al., 2000), several genes involved in melanin biosynthesis have been isolated, such as *PKS1*, *THR1*, and *SCD1*. *CgPKS1*, a melanin biosynthesis gene in *C. graminicola*, is not required for appressoria turgor generation, but is important for penetration and melanin synthesis (Ludwig et al., 2014). In this study, the Δ*CgMK1* mutants exhibited slightly darkened colonies. The expression of three melanin biosynthesis genes including *PKS1*, *THR1*, and *SCD1* were significantly down-regulated in the Δ*CgMK1* mutants.

Previous studies have demonstrated that deletion of the Fus3/Kss1-related pathway results in a loss of pathogenesis owing to an inability of several pathogenic fungi to colonize host tissues or form infection structures, for example *VMK1* in *V. dahliae*, and *PMK1* in *M. oryzae* (Xu and Hamer, 1996). Knockout of *CgMK1* resulted in mutants that were non-pathogenic and unable to form lesions on poplar leaves. On the poplar epidermis, Δ*CgMK1* mutants formed elongated germ tubes, but were unable to infect the epidermal cells. In addition, penetration assays with cellophane membranes and onion epidermal cells suggested that the Δ*CgMK1* mutants were unable to penetrate the cellophane membranes. Furthermore, we discovered that the hyphae of the Δ*CgMK1* mutants could only marginally infect onion epidermal cells. This result differs from that displayed in other fungi such as *C. orbiculare* (Takano et al., 2000), where the Fus3/Kss1 ortholog mutant completely lost the ability to attack the host via the wounded sites. The results indicated that *CgMK1* is important for penetration and infectious growth once inside the plant. The relationship of Fus3/Kss1 MAPK cascade and plant immunization pathways has also been examined. A thioredoxin gene, *TRX2*, which is essential for intracellular ROS signaling, affects invasive growth via the Mst11-Mst7-PMK1 pathway (Wang et al., 2016). Recently, some genes from other families that are related to ROS and pathogenicity in *C. gloeosporioides*, such as *CgAP1* and *CgRhoB* have been identified. Deletion of *CgAP1* results enhances a strain sensitivity to oxidative stress, and a reduction in virulence (Sun et al., 2016). The Δ*CgRhoB* mutants affect cAMP levels and stress pathways, resulting in a significant reduction in poplar leaf virulence (Xu et al., 2016). Further investigations should therefore focus on elucidating the relationship between the *CgMK1* gene of *C. gloeosporioides* and host defense. Previously, upstream components of the Fus3/Kss1 MAPK cascade have been identified and proved to be critical to appressorium formation and pathogenicity of *Colletotrichum*, such as CoMEKK1 of *C. orbiculare* (Sakaguchi et al., 2010) and ChSte7 in *C. higginsianum* (Yuan et al., 2016). Systematic studies of the Fus3/Kss1 signaling cascades in *C. gloeosporioides* are not well-characterized.

In summary, *CgMK1* acts as a key regulator of appressorium formation and plant infection in *C. gloeosporiodes*. Furthermore, Δ*CgMK1* mutants exhibit deficiency in invasive growth on media or onion epidermal cells. These findings improve our understanding toward the functions of the MAPK signaling pathway in *C. gloeosporioides*.

## Author Contributions

YW, CT, and PH designed the experiments. PH, XW, and XZ performed the experiments and the data analyses. YW and PH prepared the figures and wrote the manuscript.

## Conflict of Interest Statement

The authors declare that the research was conducted in the absence of any commercial or financial relationships that could be construed as a potential conflict of interest. The reviewer PP-N and handling Editor declared their shared affiliation.

## Footnotes

1 http://genome.jgi.doe.gov/Gloci1/Gloci1.home.html

2 http://www.ebi.ac.uk/interpro/

## Supplementary Material

FIGURE S1 Phylogenetic analysis of *CgMK1* and other homologs from other fungi. **(A)** Prediction of domains in *CgMK1* used the InterPro website (http://www.ebi.ac.uk/interpro/). Amino-acid sequences of *CgMK1* in *Colletotrichum gloeosporioides* and Fus3/Kss1 homologs in other fungi were aligned by the ClustalX 2.1. The conserved TEY sequence needed for kinase activation is marked by asterisks. aa, amino acid. **(B)** Phylogenetic tree of *CgMK1* and its homologs in *Magnaporthe oryzae* (PMK1, AAC49521), *Gaeumannomyces graminis* (GMK1, AAG44657), *Fusarium graminearum* (GPMK1, AAL73403), *Nectria haematococca* (FsMAPK, Q00859), *Colletotrichum orbiculare* (CMK1, AAD50496), *Mycosphaerella graminicola* (MgFus3, XP_003851863), *Aspergillus nidulans* (AnFUS3, AN3719), *Fusarium oxysporum* (FMK1, AAG01162), *Botrytis cinerea* (BMP1, AAG23132). The phylogenetic tree was constructed by MEGA 7.0 with full-length protein sequences and neighbor-joining with 1000 bootstrap replicates.

FIGURE S2 Disruption and complementation of *CgMK1* in *C. gloeosporioides*. **(A,B)** Replacement method for the deletion of *CgMK1*. The 1.0 kb sequence of *CgMK1* was substituted for a sequence including the hygromycin cassette. Filtration of the Δ*CgMK1* mutants with primers External-CgMK1for and External-CgMK1rev **(A)** and primers RT-CgMK1for and RT-CgMK1rev **(B)**. **(C)** Confirmation of the Δ*CgMK1* mutants and complementation of *CgMK1* by semiquantitative reverse transcription PCR. **(D,E)** Southern blot of wild-type strain and two independent Δ*CgMK1* mutants (Δ*CgMK1-8*, Δ*CgMK1-13*). DNA specimens of transformants were digested by *Sca*I. The enzyme-digested product was probed with a DNA sequence from a hph gene (Probe 3′ of the HPH) **(D)** and *CgMK1* (A 493 bp fragment of the 3′flanking sequence of *CgMK1*) **(E)**.

FIGURE S3 Sensitivity of the *CgMK1* mutants to cell wall integrity stresses. **(A)** Colony morphology of the wild-type strain, Δ*CgMK1* mutants (Δ*CgMK1-8*, Δ*CgMK1-13*) and complemented strain after 3 days of growth on CM or CM containing congo red, CFW. **(B)** The bar chart showed the colony diameter of wild-type, mutants and complementation strain under different chemical stresses. Data sets were calculated from the picture **(A)**. “a” and “b” indicate a significant difference between wild-type, mutant and complementation strain at *P* = 0.05 according to Duncan’s range test.
