# Supplementary figures and images for: The Mitogen-Activated Protein Kinase CgMK1 Governs Appressorium Formation, Melanin Synthesis, and Plant Infection of Colletotrichum gloeosporioides
Source: Front Microbiol. 2017 Nov 10;8:2216. doi: 10.3389/fmicb.2017.02216 (PMC5686099; doi:10.3389/fmicb.2017.02216)

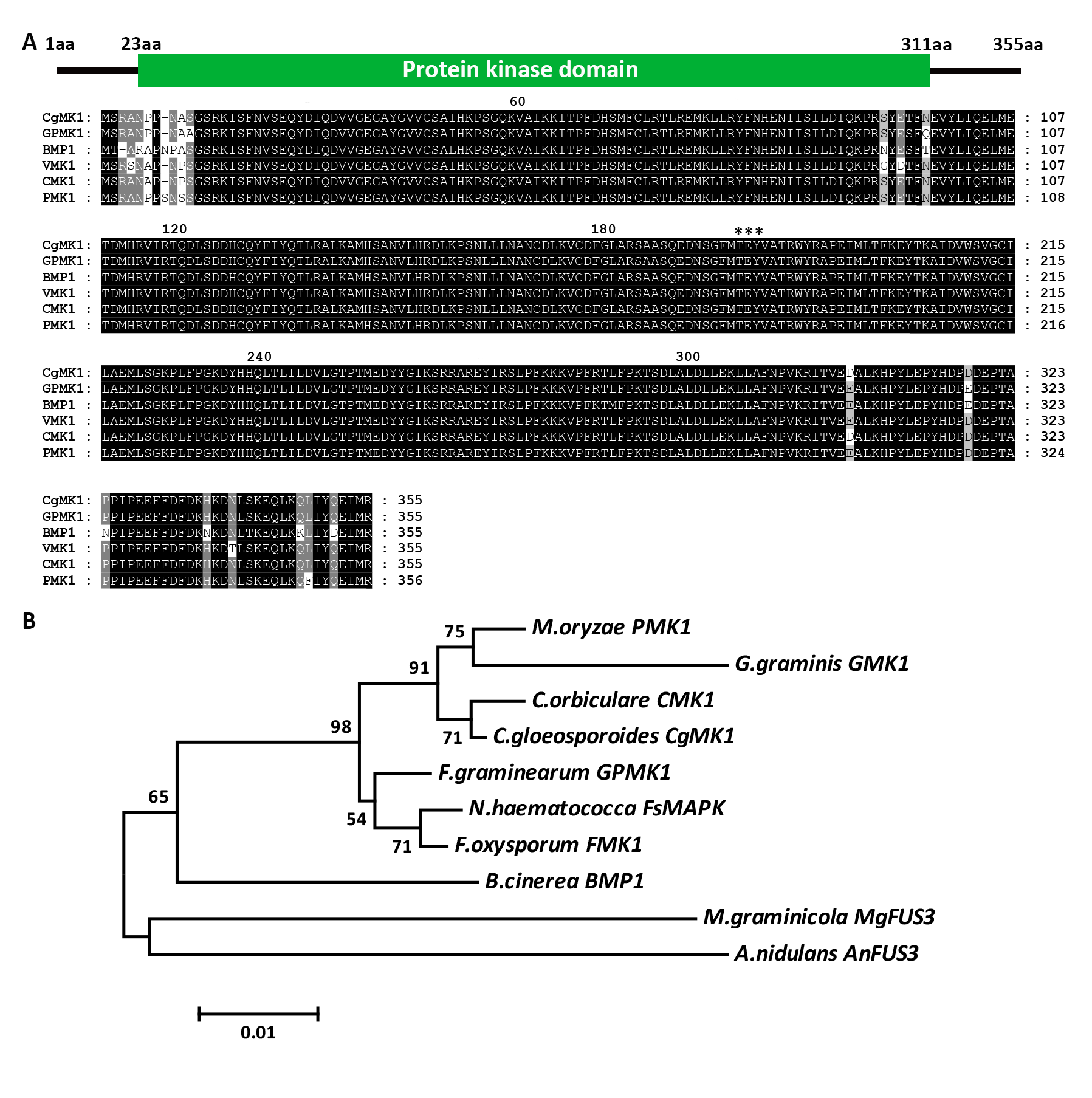

Supplement: FIGURE S1 — Phylogenetic analysis of CgMK1 and other homologs from other fungi. (A) Prediction of domains in CgMK1 used the InterPro website (http://www.ebi.ac.uk/interpro/). Amino-acid sequences of CgMK1 in Colletotrichum gloeosporioides and Fus3/Kss1 homologs in other fungi were aligned by the ClustalX 2.1. The conserved TEY sequence needed for kinase activation is marked by asterisks. aa, amino acid. (B) Phylogenetic tree of CgMK1 and its homologs in Magnaporthe oryzae (PMK1, AAC49521), Gaeumannomyces graminis (GMK1, AAG44657), Fusarium graminearum (GPMK1, AAL73403), Nectria haematococca (FsMAPK, Q00859), Colletotrichum orbiculare (CMK1, AAD50496), Mycosphaerella graminicola (MgFus3, XP_003851863), Aspergillus nidulans (AnFUS3, AN3719), Fusarium oxysporum (FMK1, AAG01162), Botrytis cinerea (BMP1, AAG23132). The phylogenetic tree was constructed by MEGA 7.0 with full-length protein sequences and neighbor-joining with 1000 bootstrap replicates. [file Image_1.TIF]

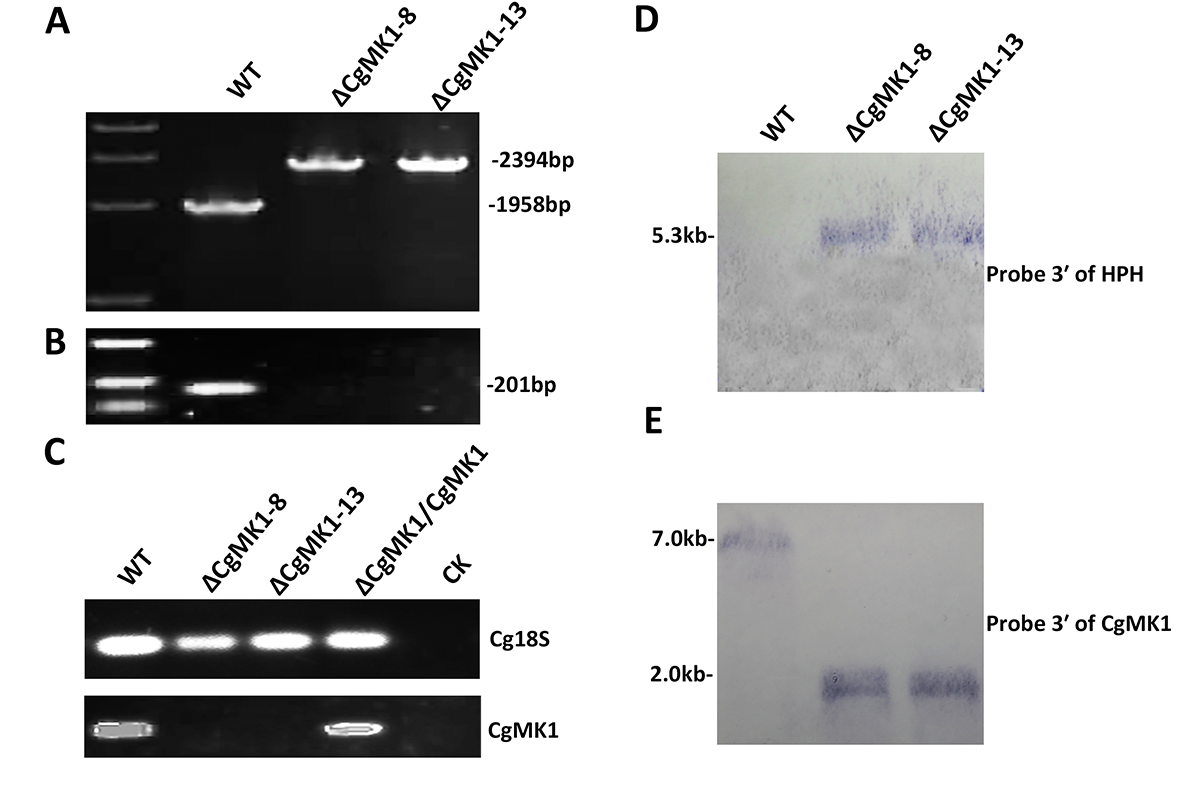

Supplement: FIGURE S2 — Disruption and complementation of CgMK1 in C. gloeosporioides. (A,B) Replacement method for the deletion of CgMK1. The 1.0 kb sequence of CgMK1 was substituted for a sequence including the hygromycin cassette. Filtration of the ΔCgMK1 mutants with primers External-CgMK1for and External-CgMK1rev (A) and primers RT-CgMK1for and RT-CgMK1rev (B). (C) Confirmation of the ΔCgMK1 mutants and complementation of CgMK1 by semiquantitative reverse transcription PCR. (D,E) Southern blot of wild-type strain and two independent ΔCgMK1 mutants (ΔCgMK1-8, ΔCgMK1-13). DNA specimens of transformants were digested by ScaI. The enzyme-digested product was probed with a DNA sequence from a hph gene (Probe 3′ of the HPH) (D) and CgMK1 (A 493 bp fragment of the 3′flanking sequence of CgMK1) (E). [file Image_2.TIF]

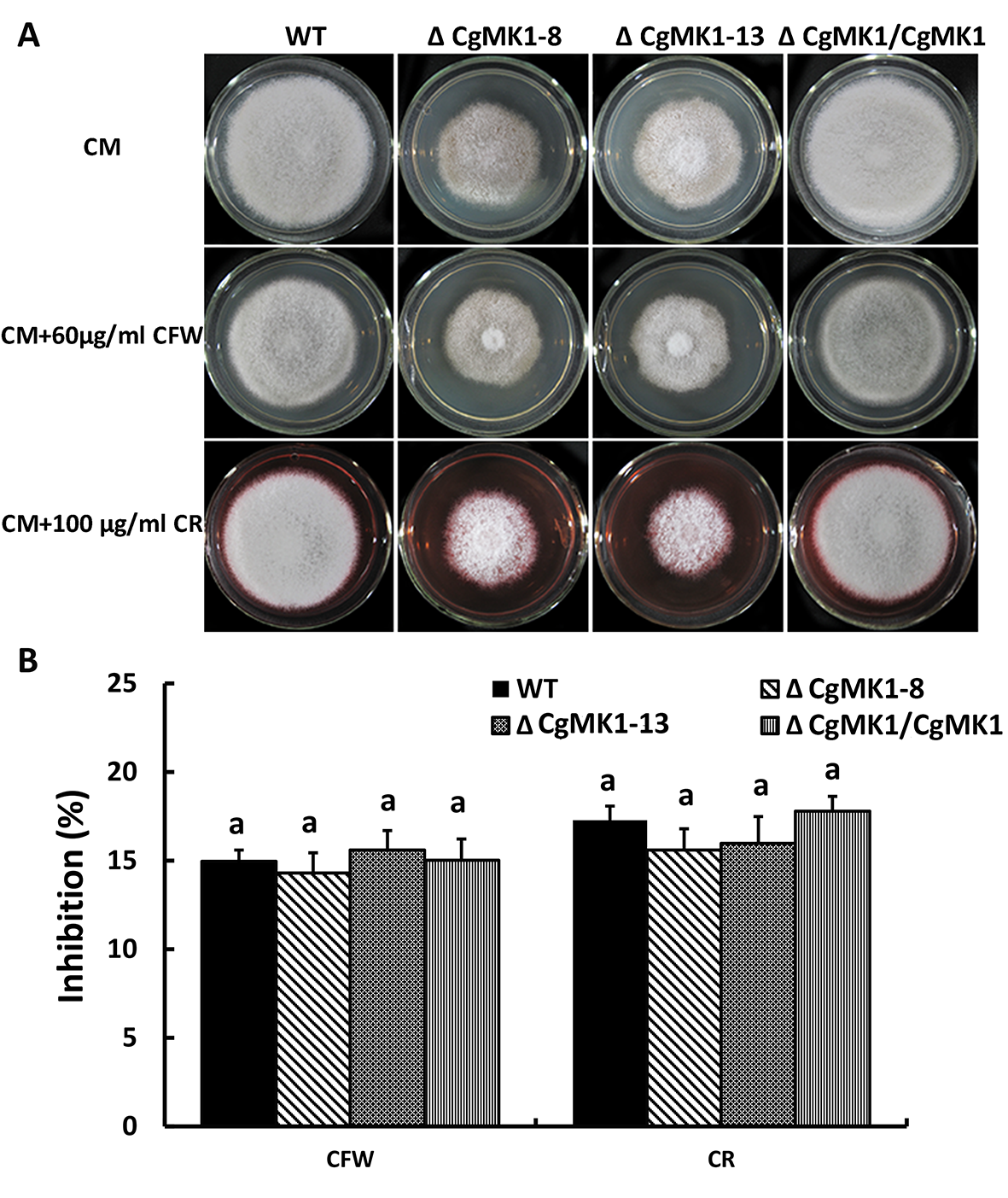

Supplement: FIGURE S3 — Sensitivity of the CgMK1 mutants to cell wall integrity stresses. (A) Colony morphology of the wild-type strain, ΔCgMK1 mutants (ΔCgMK1-8, ΔCgMK1-13) and complemented strain after 3 days of growth on CM or CM containing congo red, CFW. (B) The bar chart showed the colony diameter of wild-type, mutants and complementation strain under different chemical stresses. Data sets were calculated from the picture (A). “a” and “b” indicate a significant difference between wild-type, mutant and complementation strain at P = 0.05 according to Duncan’s range test. [file Image_3.TIF]
